# Sulfonamide-Based Inhibition of the β-Carbonic Anhydrase from *A. baumannii*, a Multidrug-Resistant Bacterium

**DOI:** 10.3390/ijms252212291

**Published:** 2024-11-15

**Authors:** Viviana De Luca, Simone Giovannuzzi, Clemente Capasso, Claudiu T. Supuran

**Affiliations:** 1 Department of Biology, Agriculture and Food Sciences, National Research Council (CNR), Institute of Biosciences and Bioresources, 80131 Naples, Italy; viviana.deluca@ibbr.cnr.it; 2 Neurofarba Department, Section of Pharmaceutical Sciences, University of Florence, Via Ugo Schiff 6, Sesto Fiorentino, 50019 Florence, Italy; simone.giovannuzzi@unifi.it (S.G.); claudiu.supuran@unifi.it (C.T.S.)

**Keywords:** carbonic anhydrase, *Acinetobacter baumannii*, pathogens, sulfonamide inhibitors

## Abstract

*Acinetobacter baumannii* is a Gram-negative opportunistic pathogen responsible for severe hospital-associated infections. Owing to its ability to develop resistance to a wide range of antibiotics, novel therapeutic strategies are urgently needed. One promising approach is to target bacterial carbonic anhydrases (CAs; EC 4.2.1.1), which are enzymes critical for various metabolic processes. The genome of *A. baumannii* encodes a β-CA (βAbauCA), which is essential for producing bicarbonate ions required in the early stages of uridine triphosphate (UTP) synthesis, a precursor for the synthesis of peptidoglycans, which are vital components of the bacterial cell wall. This study aimed to inhibit βAbauCA in vitro, with the potential to impair the vitality of the pathogen in vivo. We conducted sequence and structural analyses of βAbauCA to explore its differences from those of human CAs. Additionally, kinetic and inhibition studies were performed to investigate the catalytic efficiency of βAbauCAβ and its interactions with sulfonamides and their bioisosteres, classical CA inhibitors. Our results showed that βAbauCA has a turnover rate higher than that of hCA I but lower than that of hCA II and displays distinct inhibition profiles compared to human α-CAs. Based on the obtained data, there are notable differences between the inhibition profiles of the human isoforms CA I and CA II and bacterial βAbauCA. This could open the door to designing inhibitors that selectively target bacterial β-CAs without affecting human α-CAs, as well as offer a novel strategy to weaken *A. baumannii* and other multidrug-resistant pathogens.

## 1. Introduction

*Acinetobacter baumannii* is a Gram-negative opportunistic pathogen that causes severe infections, particularly in hospital settings. It is often associated with conditions such as ventilator-associated pneumonia, bloodstream infections, and wound infections, particularly in immunocompromised individuals or in those with invasive medical devices [1,2,3,4]. *A. baumannii* can resist a wide range of therapeutic agents, including aminoglycosides, beta-lactams, carbapenems, cephalosporins, and tetracyclines [5,6]. The bacterium has developed several sophisticated mechanisms to evade antibiotic action, making it particularly challenging to treat [7,8,9,10]. These mechanisms include the modification of membrane permeability, the active efflux of antibiotics, enzymatic inactivation, target-site alterations, and biofilm formation [5,6]. Additionally, the high genetic plasticity of *A. baumannii*, facilitated by horizontal gene transfer and the acquisition of mobile genetic elements, contributes significantly to its ability to rapidly evolve resistance [11].

The increasing prevalence of multidrug-resistant (MDR) *A. baumannii* strains often results in prolonged hospital stays, increased mortality rates, and higher healthcare costs [7,8,9,10]. A multifaceted strategy has been employed to combat *A. baumannii* infections using combination antibiotic therapies, infection control measures (e.g., strict hygiene and isolation protocols), and the development of novel antimicrobial agents. Consequently, research has been focused on targeting the resistance mechanisms of *A. baumannii* [12]. For example, bacterial efflux pumps have been targeted, which expel antibiotics from the cell and thereby reduce their effectiveness [13,14]. Efforts are also directed at inhibiting carbapenemases and beta-lactamases, enzymes that break down essential antibiotics, such as carbapenems and beta-lactams [15,16,17]. Additionally, research has examined porin channels that impede antibiotic entry and biofilm formation, which protects bacteria from antibiotic treatment [18,19]. Alternative treatments, such as bacteriophage and immune-based therapies, are also being explored [20,21,22]. 

One such innovative approach is to target bacterial enzymes that play pivotal roles in bacterial survival, virulence, and pathogenesis. Among these, carbonic anhydrases (CAs, EC 4.2.1.1), enzymes involved in maintaining pH balance and facilitating critical metabolic processes, have emerged as promising new targets. CAs are metalloenzymes found in both eukaryotic and prokaryotic organisms that catalyze the reversible hydration of carbon dioxide (CO_2_) into bicarbonate (HCO_3_^−^) and protons (H^+^) [23,24,25,26,27,28]. In bacteria, CAs provide adaptability and resilience, particularly under the stressful conditions encountered during host infection [23,29,30,31,32,33,34]. In addition, bicarbonate is an essential metabolite in various biosynthetic pathways, including those involved in fatty acid and nucleotide synthesis [35]. These pathways are critical for bacterial growth and replication, particularly under nutrient-limited or stressful conditions such as those encountered during infection. Consequently, the inhibition of bacterial CAs could disrupt these critical processes, making them a valuable target for antimicrobial strategies. In particular, the *A. baumannii* genome encodes for a β-CA (βAbauCA or CanB), which provides essential bicarbonate ions required for the early stages of uridine triphosphate (UTP) synthesis. UTP is critical to produce key cellular components, such as UDP-N-acetylglucosamine, a precursor in the synthesis of peptidoglycans, which are fundamental components of the bacterial cell wall [36,37]. By supporting these biosynthetic pathways, βAbauCA helps to maintain the bacterium’s viability. Bacterial CAs are structurally and functionally different from their human counterparts, making it possible to develop selective inhibitors that specifically target bacterial enzymes without affecting CAs of the host [38,39,40]. Classical carbonic anhydrase inhibitors (CAIs), such as sulfonamides and their biososters, have been widely studied for their ability to inhibit CAs in both the human and bacterial systems [40]. By focusing on this enzyme, we can target a mechanism that does not overlap with pathways typically affected by conventional antibiotics, thereby reducing the risk of cross-resistance. The use of CAIs in combination with antibiotics forms part of a broader strategy to avoid the development of further bacterial resistance to CAIs [41,42,43]. CAIs do not directly kill bacteria but instead weaken critical processes such as pH regulation, bicarbonate metabolism, and stress responses, making the pathogen more susceptible to antibiotics [29]. 

In this context, this manuscript delves into the potential of using sulfonamides and their bioisosteres recognized for their role as CAIs to target the βAbauCA. This enzyme has been previously successfully cloned and characterized by our research groups [36]. Here, the βAbauCA inhibition profile has been compared with those obtained with the two human α-CA isoforms (hCA I and hCA II) with the objective of identifying promising highly selective inhibitors for the bacterial enzyme. Targeting βAbauCA could not only provide a new strategy to impair the bacterial growth, but also open the door to more precise and effective treatments for *A. baumannii* and other multidrug-resistant pathogens.

## 2. Results and Discussion

### 2.1. Sequence and Structural Analysis of βAbauCA

**Figure 1:**
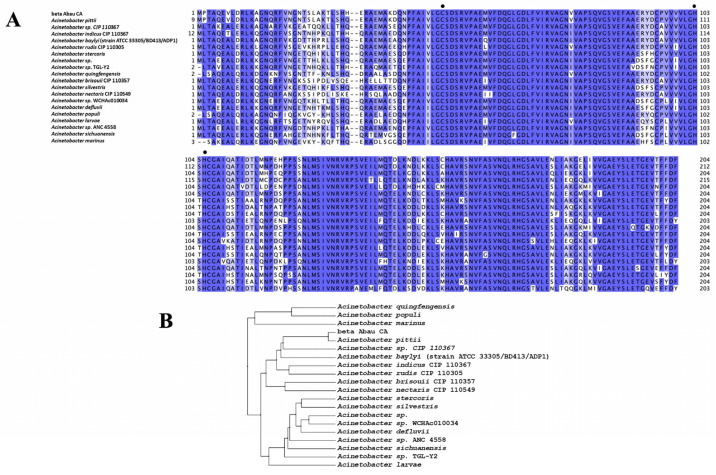
Multiple sequence alignment and dendrogram of β-CAs in the Acinetobacter genus. (**A**). The multiple sequence alignment of β-CAs belonging to various species within the Acinetobacter genus was performed with the program MUSCLE to identify conserved residues critical for enzyme function. The alignment highlights key amino acid residues involved in the coordination of the zinc ion (indicated with black dots) and reveals the conservation of essential residues (blue color) across different Acinetobacter species. Uncolored amino acid residues represent variations in the sequences considered. (**B**). The dendrogram, generated using PhyML 3.0 to create a bootstrap consensus tree with 100 replicates, illustrates the genetic relationships among carbonic anhydrases from various Acinetobacter species based on their amino acid sequences. The clustering of β-AbauCA with other Acinetobacter CAs indicates a shared evolutionary history and functional similarity. In contrast, distinct clusters formed by certain species suggest specific adaptations to their unique ecological niches. Sequence accession numbers: *A. pittii*, WP_014206063.1; *A.* sp CIP 110367, WP_004681417.1; *A. indicus* CIP 110367: WP_035363289.1; *A. baylyi* (strain ATCC 33305/BD413/ADP1), WP_004921672.1; *A. rudis* CIP 110305, WP_016657123.1; *A. stercoris*, WP_121973703.1; *A.* sp, WP_291352560.1; *A.* sp. TGL-Y2, WP_067656738.1; *A. quingfengensis*, WP_070069596.1; *A. brisouii* CIP 110357, WP_004903844.1; *A. silvestris*, WP_086203590.1; *A. nectaris* CIP 110549, WP_023273849.1; *A.* sp WCHAc010034, WP_068907360.1; *A. defluvii*, WP_065993606.1; *A. populi*, WP_087621477.1; *A. larvae*, WP_067556740.1; *A.* sp ANC 4558, WP_086180767.1; *A. sichuanensis*, WP_107007476.1; *A. marinus*, WP_092614828.1.

β-CAs are critical enzymes in converting CO_2_ into bicarbonate, a process essential for maintaining carbon metabolism across a wide range of species. This functional significance is evident in *A. baumannii*, in which the enzyme βAbauCA demonstrates considerable similarities to β-CAs from other species within the Acinetobacter genus (Figure 1A).

**Figure 2:**
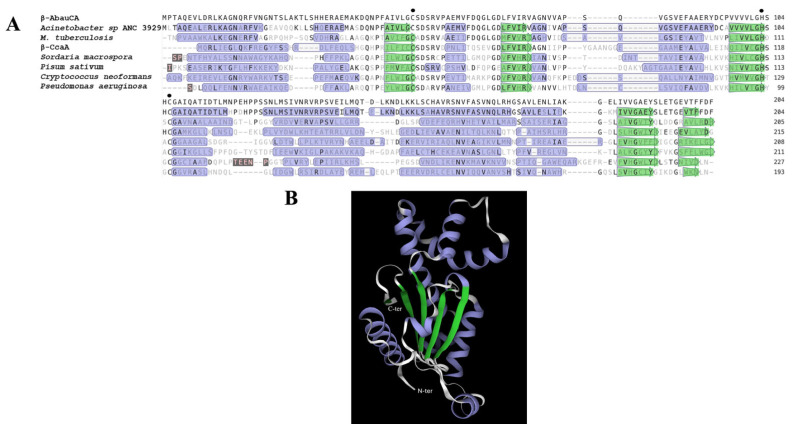
Structural modeling of βAbauCA. (**A**). Structural templates belonging to the β-CAs were identified and aligned with the βAbauCA target sequence using the SWISS-MODEL template library to generate a reliable structural model. The PDB entry of the templates are as follows: N9K418.1 (*Acinetobacter* sp. ANC 3929), 2A5V.1 (*M. tuberculosis*), 5SWC.1. (β-CcaA), 4O1J.1 (*Sordaria macrospora*), 1EKJ.1 (*Pisum sativum*), 2W3Q.1 (*Cryptococcus neoformans*), and 5JJ8.1 (*Pseudomonas aeruginosa*). (**B**). Cartoon representation depicting the overall fold of the βAbauCA model, illustrating the key secondary structural elements. Legend: Green arrows represent β-strands, purple boxes indicate α-helices, and brown boxes denote residues absent in the target sequence.

This relationship underscores the evolutionary conservation of key amino acid residues and catalytic mechanisms across the diverse Acinetobacter species. The conservation of these residues indicates their integral roles in ecological and evolutionary contexts, particularly in carbon metabolism. Two cysteine (Cys) residues and one histidine (His) residue are crucial for coordinating the Zn^2+^ ion within the active site of Acinetobacter β-CAs (Figure 1A). The presence of these residues facilitates efficient catalysis, which is essential for the conversion of CO_2_ to bicarbonate. This suggests that the catalytic mechanisms and overall functions of β-CAs are vital for the survival and adaptation of these organisms in various environmental niches. Moreover, the functional integrity of βAbauCA was reinforced by a range of highly conserved residues identified during sequence alignment (Figure 1A). This conservation across different Acinetobacter species indicates that these residues are crucial not only for enzyme stability but also for enhancing catalytic efficiency. The dendrogram constructed from the amino acid sequences of β-CAs across various Acinetobacter species provides a visual representation of the genetic relationships between these enzymes (Figure 1B). Notably, βAbauCA clustered closely with β-CAs from other Acinetobacter species. This clustering implies that *A. baumannii* and its relatives have adapted to similar biochemical pathways, allowing them to manage CO_2_ and bicarbonate levels efficiently. Conversely, species such as *A. gingfengensis*, *A. populi*, and *A. marinus* were grouped in a distinct cluster in the dendrogram (Figure 1B). This branching may reflect specific adaptations or evolutionary divergences from other Acinetobacter species, potentially influenced by the unique environmental pressures or ecological niches that these organisms occupy. This divergence suggests that while each species may have developed unique adaptations to their respective environments, they fundamentally rely on β-CAs for effective carbon metabolism. This observation prompted us to conduct a more thorough investigation of the structural characteristics of βAbauCA to deepen our understanding of its architecture and potential functional implications. To this end, we utilized the SWISS-MODEL template library, a comprehensive structural database that contains experimentally determined protein structures obtained from the Protein Data Bank (PDB) [44]. By leveraging this resource, we identified structural templates that closely matched βAbauCA, thus allowing the generation of a reliable model of the three-dimensional βAbauCA structure (Figure 2A,B). 

**Figure 3:**
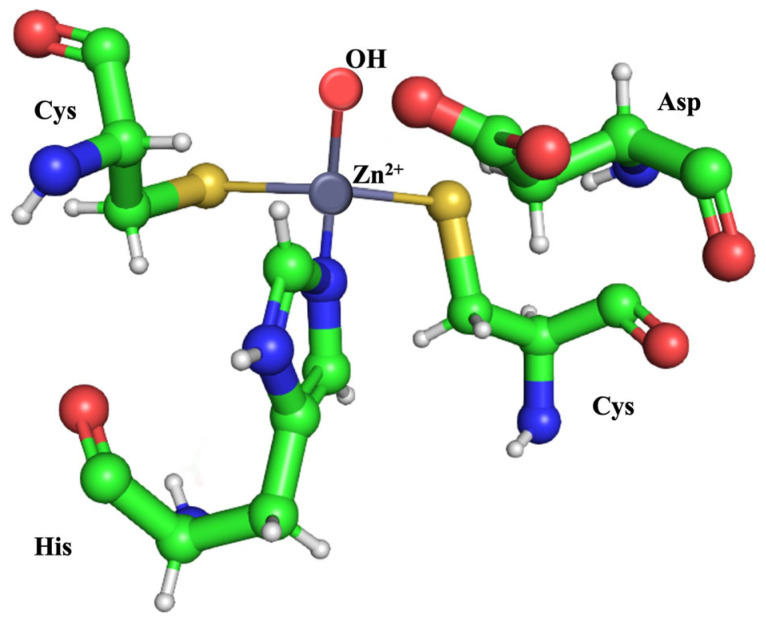
The β-CA catalytic triad. The catalytic triad consists of two cysteine residues and one histidine residue. These residues coordinate the catalytic zinc ion (Zn(II)), which is also bound to a hydroxyl ion (OH^−^) as the fourth ligand, playing a crucial role in the enzyme’s catalytic mechanism. Along with these residues, a conserved aspartate (Asp), positioned immediately after the first cysteine of the triad, is reported. However, according to the literature, this aspartate is unlikely to function as an essential fourth ligand for the active site zinc in bacterial β-CAs.

As shown in Figure 2A,B, analysis of this model revealed that βAbauCA exhibits essential architectural features that are hallmarks of classical β-carbonic anhydrases (β-CAs). Specifically, the core of the βAbauCA enzyme consists of a five-stranded β-sheet, which is encased by several α-helices, contributing to the overall stability and conformation of the protein [45] (Figure 2B). Interestingly, β-CAs have been shown to crystallize as dimers, but they can also exist in various oligomeric states, ranging from dimers to tetramers, and even octamers. The enzyme is catalytically active in its dimeric, tetrameric, and octameric forms, but is not active as a monomer [46]. In these oligomeric states, the two active sites, both originating from the same monomer and forming a coordination environment characteristic of β-CAs, are located at the dimer interface, which is a structural feature commonly observed in mesophilic β-CA homologs [45]. Additionally, in both active sites, a water molecule completes the tetrahedral coordination of the Zn(II) ion, which is essential for enzyme function. The conserved aspartate (Asp) residue positioned after the first cysteine of the catalytic triad is present [46] (Figure 2A and Figure 3). 

The human genome encodes only for the α class of CAs, while the *A. baumannii* genome encodes for a β-CA as well as for a *γ*-CA [47]. The structural alignment of α- and β-CAs presents a major challenge due to their profound structural and sequence differences, which reflect their divergent evolutionary paths. Despite catalyzing the same reaction, converting carbon dioxide to bicarbonate, and relying on zinc as their catalytic ion, these two enzyme classes have evolved distinct active site configurations and primary structures. α-CAs, such as the human hCA II, coordinate the zinc ion with three histidines in the active site [48,49]. We attempted to align the three-dimensional structure of an α-CA, such as the human hCAII, with that of a known structure of a bacterial beta-CA, such as that of *Pseudomonas aeruginosa*, using the FATCAT structural comparison tool, which is designed to identify structural similarities between proteins [50]. The FATCAT comparison was performed using the known *P. aeruginosa* β-CA structure rather than directly comparing the model of βAbauCA because the latter is a computational rather than experimentally determined structure. Using a known structure first provides a more reliable reference for evaluating structural similarities and discrepancies given the potential uncertainties in the model. 

**Figure 4:**
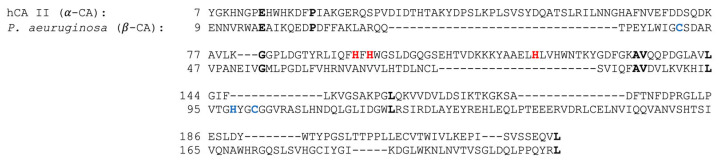
Structural alignment of α- and β-CA amino acid sequences. Using the FATCAT structural comparison tool, the three-dimensional structures of hCA II (PDB entry: 5ZXW) and bacterial CA from *P. aeruginosa* (PDB entry: 4RXY) were aligned to generate an alignment of their amino acid sequences using the FATCAT structural comparison tool. The alignment highlights identical residues in bold black along with the residues of the catalytic triads, which are colored red for α-CA and blue for the β-CA.

Despite this advanced method, the alignment results underscored the profound differences between the two enzyme classes (Figure 4). 

The FATCAT comparison revealed a very low sequence identity of just 4.63% and large structural discrepancies, with an RMSD of 4.02 Å over the 121 equivalent positions. Additionally, the alignment was marked by 138 gaps (53.28%), further highlighting the inability to align the key regions of the enzymes. Similar results were obtained using the three-dimensional model of βAbauCA, generated with SWISS-MODEL. The differences in Figure 4 reflect the distinct evolutionary paths of α- and β-CAs, which have convergently evolved to catalyze the same reaction but with radically different structural frameworks. The FATCAT results confirmed that these structural and sequence differences are not merely superficial but are deeply embedded in the protein architecture. The two structures are not significantly similar, with a *p*-value of 8.11 × 10^−1^ (structure pairs with *p*-value < 0.05 are significantly similar). α- and β-CAs both use zinc as the catalytic ion and perform the same chemical transformation, and the evolutionary paths they followed led to distinct active site architectures and amino acid sequences. This evolutionary divergence indicates that, although α- and β-CAs have convergently evolved to catalyze the same reaction, they possess distinct active site environments and primary sequences. These findings lay a strong foundation for future studies, particularly those focused on exploring the catalytic properties of enzymes and assessing their potential as a drug target. The structural differences between human and bacterial CAs create opportunities for selective inhibition, which can be leveraged to target bacterial infections without affecting human enzymes.

### 2.2. Kinetic Parameters and Inhibition Profiles

Using the stopped-flow technique and CO_2_ as the substrate, the kinetic parameters of the purified recombinant βAbauCA were determined and compared to those of the human enzymes hCA I and hCA II (Table 1). 

**Figure 5:**
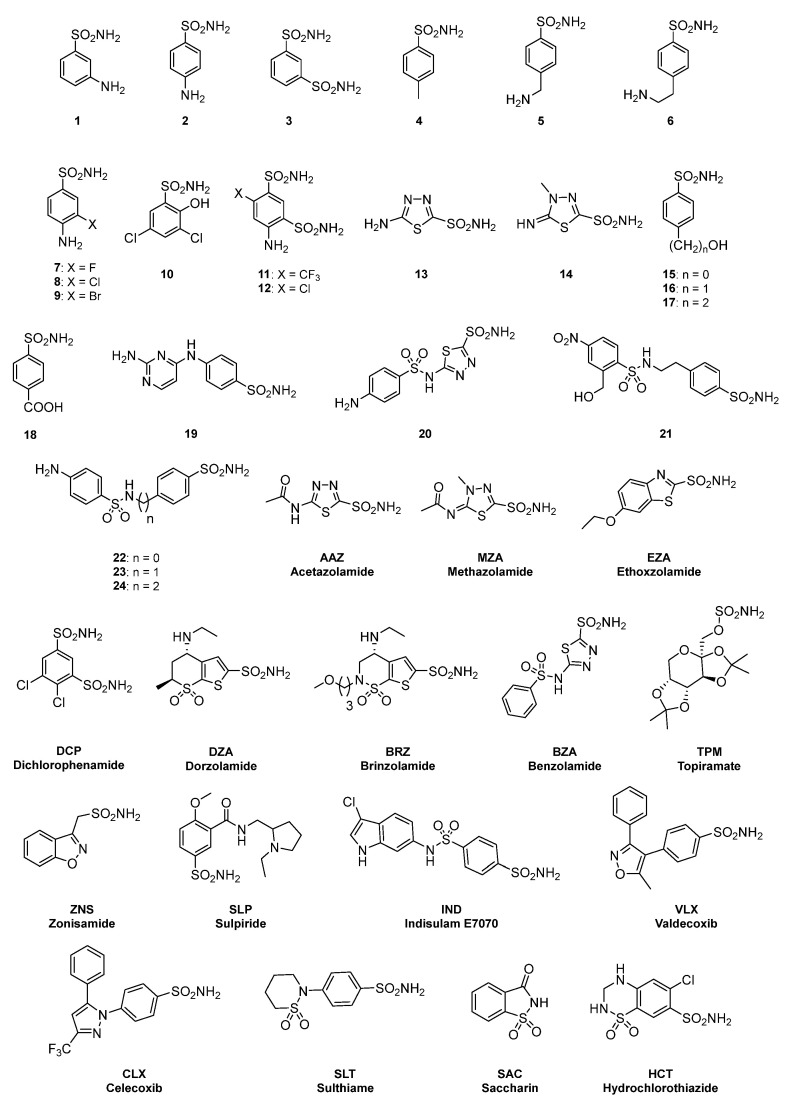
The 40 compounds used to investigate the inhibition profile of βAbauCA. The study evaluated a set of 40 compounds, consisting of 39 sulfonamides and 1 sulfamate (topiramate, TPM). These compounds are categorized into two groups: series **1–24**, and the clinically approved drugs in the **AAZ-HCT** series. This comprehensive panel was employed to assess their inhibitory effects on the bacterial βAbauCA.

The comparison shows that βAbauCA has a turnover rate (k_cat_ = 2.8 × 10^5^) which is higher than that of hCA I but significantly lower than that of hCA II. βAbauCA is less efficient than both human enzymes in terms of catalytic efficiency (k_cat_/K_m_), especially when compared to the highly efficient hCA II. These discrepancies in catalytic efficiency reflect underlying structural differences between the two enzyme classes. Although both enzyme classes interact with classical sulfonamide inhibitors through coordination with the catalytic zinc ion, the overall active-site architecture differs significantly. These differences, particularly in how the scaffold of sulfonamide inhibitors interacts with the enzyme, influence the affinity and selectivity of these inhibitors. For instance, scaffolds of sulfonamide inhibitors, such as acetazolamide (AAZ), interact differently with enzymes. This was particularly evident in the K_I_ values obtained for AAZ, which reflect the strength of these interactions. hCA I exhibited a K_I_ of 250 nM, hCA II displayed a K_I_ of 12 nM, while βAbauCA had a K_I_ of 191 nM. Therefore, we investigated the sulfonamide inhibition profile of βAbauCA using substituted benzene-sulfonamides and clinically licensed drugs. Among the carbonic anhydrase inhibitors (CAIs), a library of forty compounds (**1–24** and **AAZ-HCT**) represents an important group of simple aromatic and heterocyclic sulfonamides, including one sulfamate, which is known to effectively inhibit CAs (Figure 5). 

The **AAZ-HTC** series includes licensed drugs used for various clinical treatments, such as glaucoma, epilepsy, idiopathic intracranial hypertension, diuretics, duodenal ulcers, migraine, Parkinson’s disease, obesity, cancer, osteoarthritis, rheumatoid arthritis, and more. Most sulfonamides bind to the zinc ion (Zn(II)) in a tetrahedral geometry in their deprotonated state. This binding establishes an extended network of hydrogen bonds with the amino acid residues in the catalytic site, whereas the aromatic and heterocyclic portions of the inhibitors interact with both hydrophilic and hydrophobic residues within the catalytic cavity. This intricate interaction profile underscores the potential for the selective inhibition of βAbauCA, paving the way for the development of targeted therapeutics given that CAs are recognized as valuable targets for disrupting microbial vitality and virulence. Moreover, we compared the sulfonamide inhibition profile of βAbauCA with those obtained for the two human isoforms, hCA I and hCA II, to further identify selective inhibitors that preferentially target bacterial CA without affecting human isoforms. Therefore, this in vitro investigation is a vital step in the pursuit of innovative strategies to combat antibiotic-resistant pathogens and could enhance the efficacy of existing treatment regimens. From the data presented in Table 2, the following observations can be made:

*(a)*  *Efficient Inhibitors for hCA I and hCA II*

hCA I: Inhibitors with inhibition constants (K_I_) as low as 6.0 nM demonstrate high potency against this isoform, suggesting that certain compounds can effectively bind and inhibit hCA I at very low concentrations.

hCA II: Even more potent inhibition was observed for hCA II, with some compounds showing K_I_ values as low as 2.0 nM. This underscores the high affinity of these inhibitors for hCA II, which make them particularly efficient in targeting this isoform.

*(b)*  *Moderate Inhibition of βAbauCA*

In contrast, βAbauCA showed a wider range of K_I_ values, from 76.9 nM to 874.8 nM. This suggests moderate inhibition, with βAbauCA being less sensitive to these compounds compared to hCA I and hCA II. Although some compounds showed relatively lower K_I_ values (e.g., 76.9 nM), indicating moderate inhibition, the bacterial enzyme was less susceptible overall. Specifically, acetazolamide (**AAZ**), Ethoxzolamide (**EZA**), Brinzolamide (**BRZ**), and Benzolamide (**BZA**), including a heteroaromatic sulfonamide as functional ZBG, exhibited the most promising inhibition profiles, with K_I_ values of 191.0 nM, 76.9 nM, 170.2 nM, and 112.6 nM, respectively. Additionally, Indapamide (**IND**), which features a benzenesulfonamide group, showed a K_I_ value in the mid-nanomolar range, comparable to that of **EZA**, the most potent inhibitor against βAbauCA. Based on these results, heteroaromatic sulfonamides appear to be more effective inhibitors than benzene-sulfonamide and other ZBGs.

*(c)*  *Weak Inhibitors for βAbauCA*

Higher K_I_ values, particularly those close to 874.8 nM for βAbauCA, indicate that many compounds are weaker inhibitors of this bacterial enzyme. This is in stark contrast to human isoform II, where potent inhibition is observed at much lower concentrations. However, in comparison to hCA I, compounds **1**, **2**, **4–17**, as well as **SAC,** were more effective against the βAbauCA. Notably, among them, compounds **1**, **2**, **4–6** resulted in being 40-fold more potent against βAbauCA than hCA I. These findings highlight that the bacterial enzyme is generally more resistant to inhibition by these compounds than hCA II, and only occasionally more resistant than hCA I.

*(d)*  *Lack of Selective Inhibitors for βAbauCA*

A key goal in inhibitor development is to identify compounds that selectively target bacterial enzymes over their human isoforms. The data suggest that most of the assessed inhibitors were more effective against hCA II, as reflected by their lower K_I_ values. However, the majority showed the stronger inhibition of βAbauCA compared to cytosolic hCA I. Ideally, selective inhibitors of βAbauCA should exhibit lower K_I_ values for this bacterial enzyme while being less effective against all human isoforms. However, based on the current data, such selectivity was not achieved, as βAbauCA generally required higher inhibitor concentrations for effective inhibition.

The observed differences between α- and β-CAs open the door for designing inhibitors that selectively target bacterial β-CAs without affecting human α-CAs. Selectivity is crucial for therapeutic applications, as it allows the inhibition of bacterial carbonic anhydrases, which play essential roles in microbial survival and pathogenesis, without causing side effects linked to the inhibition of human CAs. The distinct active site structures of α-CAs and β-CAs suggest that it is possible to develop inhibitors that specifically fit the active site of bacterial β-CAs. Selective inhibition can be achieved by designing molecules that exploit the unique features of the β-CA active site, such as differences in amino acid residues or active site conformation. Small modifications in the inhibitor structure, such as altering the functional groups or side chains, could enhance the binding affinity for β-CAs while reducing interactions with α-CAs. 

## 3. Materials and Methods

### 3.1. Chemicals and Instruments

All chemicals used in this study were of reagent grade and sourced from Sigma, Kawasaki-shi, Japan. The His-Trap FF affinity column and the AKTA-Prime purification system were acquired from GE Healthcare, Chicago, IL, USA. The SX20 Stopped-Flow apparatus was obtained from Applied Photophysics, Leatherhead, UK.

### 3.2. Cloning, Expression, and Purification of Recombinant βAbauCA

The synthetic gene encoding βAbauCA was designed and synthesized by Invitrogen GeneArt (ThermoFisher Scientific, Waltham, MA, USA), a company specializing in gene synthesis. This gene was cloned into the expression vector pET100D-Topo/βAbauCA. The construct was designed to produce recombinant βAbauCA as a fusion protein with a His-tag (six histidine residues) at the N-terminus. Competent *E. coli* BL21 (DE3) cells (Agilent, Santa Clara, CA, USA) were transformed as previously described. Protein expression was induced with 1 mM isopropyl β-D-1-thiogalactopyranoside (IPTG). Following cell growth, the cells were harvested and lysed via sonication.

Purification of the recombinant βAbauCA was performed using a nickel affinity column (His-Trap FF) designed to capture His-tagged proteins through interactions with Ni^2+^ ions. The HisTrap column (1 mL) was equilibrated with 20 mL of equilibration buffer (50 mM Tris, 20 mM imidazole, and 150 mM sodium chloride, pH 7.5) at a flow rate of 1 mL/min. The supernatant from the cell lysate was then loaded onto the column at 1 mL/min, and the bound protein was eluted using 300 mM imidazole at a flow rate of 0.5 mL/min in a buffer containing 50 mM Tris and 300 mM sodium chloride, pH 7.5. The eluted βAbauCA was determined to be 90% pure, and protein quantification was performed using the Bradford assay (Bio-Rad, Hercules, CA, USA) [51]. h CAI and hCA II were recombinant enzymes obtained in-house. The purity of both CA I and CA II proteins is high (>90%) and consistent, ensuring reliable comparison of their activities.

### 3.3. Kinetic Parameters and Inhibition Constants

The CO_2_ hydration activity of CAs was assessed using an Applied Photophysics stopped-flow spectrophotometer [52,53]. Phenol red (0.2 mM) served as the pH indicator, with absorbance measured at a maximum of 557 nm. The stopped-flow approach can sense pH-driven changes in the UV signals by measuring how the dye’s color (UV absorbance) shifts in response to pH changes, allowing us to track the enzyme’s function in a dynamic system. The reactions for isoforms hCA I and hCA II (α-CAs) were conducted at 20 °C in 10 mM HEPES buffer (pH 7.5). For βAbauCA, reactions were performed at 20 °C in 20 mM TRIS buffer (pH 8.3), supplemented with 20 mM NaClO_4_ to maintain a constant ionic strength. The initial rates of the carbonic anhydrase-catalyzed CO_2_ hydration reactions were recorded over a period of 10–100 s. Kinetic parameters were derived by constructing Lineweaver–Burk plots, using CO_2_ concentrations ranging from 1.7 to 17 mM. For each inhibitor, the initial reaction velocity was determined from at least six measurements, covering the 5–10% reaction range. The rates of uncatalyzed reactions were also measured and subtracted from the total observed rates. Inhibitor stock solutions (10–100 mM) were prepared in distilled–deionized water and further diluted to as low as 0.01 mM in the assay buffer. The enzyme and inhibitor mixtures were preincubated at room temperature for 15 min to allow for enzyme–inhibitor complex formation or to facilitate any potential hydrolysis of the inhibitor within the active site. Inhibition constants (K_I_) were calculated using non-linear least-squares regression with PRISM 6 software, applying the Cheng–Prusoff equation [54,55]. The reported values represent the means of at least three independent determinations. 

### 3.4. Phylogenetic Analysis

Phylogenetic analysis was conducted using the NGPhylogeny.fr webservice (https://ngphylogeny.fr, accessed on 11 September 2024), which offers a comprehensive suite of tools tailored for various levels of user expertise in phylogenetic studies. Multiple sequence alignment was performed using the MUSCLE (Multiple Sequence Comparison by Log-Expectation) algorithm, which is known for its speed and accuracy in generating alignments [56]. Phylogenetic trees were constructed utilizing the Neighbor-Joining method for phylogenetic analysis. A bootstrap analysis was performed to assess the reliability of the constructed phylogenetic tree [57].

### 3.5. Model Generation

The three-dimensional model of β-AbauCA was generated using the SWISS-MODEL server (https://swissmodel.expasy.org, accessed on 25 September 2024) [44]. This server employs homology modeling techniques, utilizing known protein structures as templates to predict the structure of the target protein.

### 3.6. Sequence-Structure Alignment

The sequence-structure alignment was generated using the FATCAT tool (https://fatcat.godziklab.org, accessed on 25 September 2024) [50]. This method combines structural information with sequence data to provide a more accurate alignment. The structural alignment process in FATCAT is based on the chaining of aligned fragment pairs (AFPs) while allowing a specified number of twists (t). To facilitate a rigid structure alignment, the number of twists can be constrained to zero. The algorithm employs dynamic programming techniques to determine the optimal chaining of AFPs, thereby enhancing the accuracy of the flexible alignment.

## 4. Conclusions

The sequence and structural analysis of β-CA from *A. baumannii* highlights both evolutionary conservation and key differences when compared to α-CAs, such as those found in humans. βAbauCA, like other β-CAs, plays a crucial role in the conversion of CO_2_ to bicarbonate, which is an essential process for carbon metabolism. The alignment of β-CAs from various *Acinetobacter* species showed conserved residues critical for the coordination of zinc ions, which are essential for enzymatic catalysis. These conserved residues suggest that β-CAs have evolved to maintain efficient catalysis across different species, supporting their survival in various environmental niches. Structurally, βAbauCA exhibits hallmark features of β-CAs, such as core β-sheet architecture surrounded by α-helices and a conserved catalytic mechanism involving zinc coordination by two cysteine residues and one histidine residue. This architecture, which is crucial for catalytic efficiency, differs significantly from that of α-CAs, which use three histidine residues to coordinate zinc. The structural analysis of βAbauCA, generated using the SWISS-MODEL template library, further confirmed these differences. The FATCAT alignment between α- and β-CAs underscored substantial structural discrepancies, with a low sequence identity of 4.63%, RMSD of 4.02 Å, and 138 gaps, illustrating the profound evolutionary divergence between the two classes. Despite these structural differences, both α- and β-CAs catalyze the same CO_2_ hydration reaction. However, the distinct active-site configurations of βAbauCA suggest opportunities for selective inhibition. This was demonstrated through kinetic studies and inhibition profiles comparing βAbauCA with hCA I and hCA II. βAbauCA showed a catalytic efficiency (k_cat_/K_m_) lower than that of hCA II but comparable to that of hCA I. Furthermore, inhibition studies with acetazolamide (AAZ) and a library of 40 sulfonamides revealed that βAbauCA interacts differently with classical sulfonamide inhibitors, as reflected by the inhibition constant (K_I_) values, suggesting differential inhibitor binding because of the distinct active site environment. 

These findings highlight the structural and functional divergence between α- and β-CAs despite their shared catalytic roles. The differences in the active site architecture and inhibitor profiles offer promising avenues for the development of selective inhibitors targeting bacterial β-CAs, which could be used to combat infections caused by antibiotic-resistant *A. baumannii*. This work lays a strong foundation for future studies offering insights into the development of novel therapeutic strategies. 

## Figures and Tables

**Table 1:** Kinetic parameters for the CO_2_ hydration reaction catalyzed by the α- and β-CAs: hCA I and II (α-CAs) at 20 °C and pH 7.5 in 10 mM HEPES buffer; βAbauCA determined at 20 °C, pH 8.3 in 20 mM TRIS buffer and 20 mM NaClO_4_. Inhibition data for the clinically used sulfonamide AAZ (5-acetamido-1,3,4-thiadia-zole-2-sulphonamide) are also provided.

Organism	Acronym	k_cat_ (s^−1^)	k_cat_/K_m_ (M^−1^ × s^−1^)	K_I_AAZ (nM)
*Homo sapiens*	hCA I	2.0 × 10^5^	5.0 × 10^7^	250
	hCA II	1.4 × 10^6^	1.5 × 10^8^	12
*A. baumannii*	βAbauCA	2.8 × 10^5^	1.5 × 10^7^	191

Mean from 3 different assays by a stopped-flow technique (errors were in the range of ±5–10% of the reported values).

**Table 2:** Inhibition of human isoforms hCA I and hCA II and the bacterial enzyme βAbauCA with sulfonamides **1–24** and the clinically used drugs **AAZ-HCT**, by a CO_2_ hydrase, stopped-flow assay.

Name	K_I_ (nM) ^a^		
	CA I	CA II	βAbauCA
**1**	28,000	300	678.4
**2**	25,000	240	650.1
**3**	79.0	8.0	609.1
**4**	78,500	320	1484
**5**	25,000	170	549.1
**6**	21,000	160	533.7
**7**	8300	60.0	412.7
**8**	9800	110	747.4
**9**	6500	40.0	923.7
**10**	7300	54.0	4160
**11**	5800	63.0	928.4
**12**	8400	75.0	1205
**13**	8600	60.0	443.5
**14**	9300	19.0	603.8
**15**	5500	80.0	624.8
**16**	9500	94.0	699.7
**17**	21,000	125	714.7
**18**	164	46.0	547.4
**19**	109	33.0	406.7
**20**	6.0	2.0	343.8
**21**	69.0	11.0	250.0
**22**	164	46.0	275.6
**23**	109	33.0	316.9
**24**	95.0	30.0	326.1
**AAZ**	250	12.0	191.0
**MZA**	50.0	14.0	248.7
**EZA**	25.0	8.0	76.9
**DCP**	1200	38.0	472.4
**DZA**	50,000	9.0	232.2
**BRZ**	45,000	3.0	170.2
**BZA**	15.0	9.0	112.6
**TPM**	250	10.0	2766
**ZNS**	56.0	35.0	2340
**SLP**	1200	40.0	6859
**IND**	31.0	15.0	88.7
**VLX**	54,000	43.0	214.6
**CLX**	50,000	21.0	269.9
**SLT**	374	9.0	541.9
**SAC**	18,540	5959	7495
**HCT**	328	290	874.8

^a^ Mean from 3 different assays, by a stopped-flow technique (errors were in the range of 5–10% of the reported values).

## Data Availability

The original contributions presented in this study are included in the article. Further inquiries can be directed to the corresponding author(s).
